# Treatment of NiMoO_4_/nanographite nanocomposite electrodes using flexible graphite substrate for aqueous hybrid supercapacitors

**DOI:** 10.1371/journal.pone.0254023

**Published:** 2021-07-02

**Authors:** Shahrzad Arshadi Rastabi, Rasoul Sarraf-Mamoory, Ghadir Razaz, Nicklas Blomquist, Magnus Hummelgård, Håkan Olin

**Affiliations:** 1 Department of Materials Engineering, Tarbiat Modares University, Tehran, Iran; 2 Department of Natural Sciences, Mid Sweden University, Sundsvall, Sweden; University of Wollongong, AUSTRALIA

## Abstract

The cycling performance of supercapacitors sometimes becomes limited when electrode materials slough off during frequent charge–discharge cycles, due to weak bonding between the active material and the current collector. In this work, a flexible graphite foil substrate was successfully used as the current collector for supercapacitor electrodes. Graphite foil substrates were treated in different ways with different acid concentrations and temperatures before being coated with an active material (NiMoO_4_/nanographite). The electrode treated with HNO_3_ (65%) and H_2_SO_4_ (95%) in a 1:1 ratio at 24°C gave better electrochemical performance than did electrodes treated in other ways. This electrode had capacitances of 441 and 184 Fg^–1^ at current densities of 0.5 and 10 Ag^-1^, respectively, with a good rate capability over the current densities of the other treated electrodes. SEM observation of the electrodes revealed that NiMoO_4_ with a morphology of nanorods 100–120 nm long was properly accommodated on the graphite surface during the charge–discharge process. It also showed that treatment with high-concentration acid created an appropriately porous and rough surface on the graphite, enhancing the adhesion of NiMoO_4_/nanographite and boosting the electrochemical performance.

## Introduction

Supercapacitors are renewable energy storage devices that are increasingly attracting attention due to their high capacitance, high power density, long cycle life, and wide operating voltage ranges [1, 2]. Nowadays, it is essential to consider utilizing materials with sustainable and green characteristics in energy storage applications [3]. The main active material groups for energy storage applications are carbonaceous materials, metal oxides, metal hydroxides, and conducting polymers [4, 5]. Carbonaceous materials, such as activated carbon, graphene, nanographite, carbon fibers, provide large surface area with good electrical conductivity and high stability, which are crucial for supercapasitors. However, carbon-based materials give low capacitance, which is a serious barrier for enhancement of the energy density of supercapacitors [3]. Metal oxides can have a high specific capacitance due to electrochemical properties related to redox reactions, but they have low electrical conductivity that limits their capacitance [6]. Additions of highly conductive carbon materials such as graphene to metal oxides have shown improvement in the conductivity and total capacitance of metal oxides. The combination of highly conductive material and metal oxide nanoparticles produces hybrid nanocomposite, which is a promising candidate for supercapacitor applications [7, 8]. Various metal oxides such as CoMoO_4_ [9], FeMoO_4_ [10], NiCo_2_O_4_ [11], MnMoO_4_ [12], and NiMoO_4_ [13, 14] have all been used in supercapacitor electrodes. However, nickel molybdate (NiMoO_4_) is considered an especially promising material for the electrode due to its high theoretical specific capacitance, battery-type behavior, low cost, and abundance [15, 16]. To produce an electrode, active material such as NiMoO_4_/nanographite (NG) is coated on a current collector. It should be mentioned that an important feature of electrode is to be flexible and could resist well against mechanical tension, i.e. bending to retain its performance without substantial degradation [3]. Ni foam is one material used as the current collector in supercapacitors, although it is inflexible and expensive [17]. Thus, it should preferably be replaced with a cheaper and more flexible current collector material. Moreover, in general, poor adhesion of active material such as NiMoO_4_/NG to the current collector causes the electrode to slough off during electrochemical performance, resulting in unstable cycling performance [2, 18, 19]. This essential challenge needs to be resolved.

This work studies the coating of NiMoO_4_/NG nanocomposite on treated graphite current collectors. The graphite foil substrate is an environmentally friendly, flexible, and cheap current collector with high conductivity that has previously been insufficiently studied as a current collector in supercapacitors. In this research, graphite foil substrates have been modified using various acid washing treatments to increase the adhesion of NiMoO_4_/NG to the surface during the coating process. This may deepen our understanding of the sloughing off of NiMoO_4_/NG during electrochemical performance.

## Experimental

### Materials

Commercial graphite foil substrate (1 × 1 cm^2^, Sigraflex graphite foil, F02012TH; SGL Carbon, Wiesbaden, Germany) was cleaned before each experiment by peeling off the top few graphene layers using sandpaper and washing with ethanol several times. HNO_3_ (65%; VWR Chemicals, Radnor, PA, USA) and H_2_SO_4_ (95%; VWR Chemicals) were used for acid treatment of the graphite foil substrate. The NG (1.6 mg mL^–1^) was produced according to the method described by Blomquist et al. [20] using thermally expanded graphite (EXG 9840; Graphit Kropfmühl, Hauzenberg, Germany) as raw material. Nickel nitrate hexahydrate (Ni(NO_3_)_2_·6H_2_O), sodium molybdate dihydrate (Na_2_MoO_4_·2H_2_O), and potassium hydroxide (KOH) were purchased from VWR Chemicals. Cellulose (10 wt%) was prepared based on Andres et al. [21] and used as an environmentally friendly binder.

### Surface modification of graphite substrate as a current collector

Graphite foil substrates (GFSs) were treated with a solution of HNO_3_:H_2_SO_4_ (1:1) to roughen the surface and improve the adhesion of materials to the surface. Various procedures were trialed, which involved dipping the GFSs in acid of different concentrations at different temperatures. One trial used a solution with low concentrations of HNO_3_ (20%):H_2_SO_4_ (20%) at 24°C. Two trials used a solution with high concentrations of HNO_3_ (65%):H_2_SO_4_ (95%) at temperatures of 24 and 60°C, respectively. Next, GFSs were ultrasonicated for 10 min, washed with deionized (DI) water, and then dried at 60°C for 24 h. Finally, three types of GFSs were prepared: GFS treated with a low-concentration solution at 24°C, i.e., GLC24, and GFS treated with a high-concentration solution at 24 and 60°C, i.e., GHC24 and GHC60, respectively. All these GFSs were compared with pristine graphite foil (G).

### Synthesis of NiMoO_4_/nanographite hybrid nanocomposite

The NiMoO_4_/NG (NMOG) nanocomposite was synthesized by chemical precipitation. In this method, an NG suspension (1.6 mg mL^–1^) was slowly added to a solution of Ni(NO_3_)_2_·6H_2_O (5 mM) and Na_2_MoO_4_·2H_2_O (5 mM) with the aid of a magnetic stirrer. After 60 min of stirring at 80°C, a uniform mixture was produced. Finally, the NMOG was washed with DI water to remove extra ions and dried at 60°C for 24 h. The final product was annealed at 400°C for 2 h under argon flow.

### Preparation of NiMoO_4_/nanographite hybrid electrodes using graphite foil current collectors

Working electrodes were prepared by mixing NiMoO_4_/NG, conductive carbon black, and cellulose (the last as a binder) in a mass ratio of 8:1:1. The mixture was then uniformly applied to different types of graphite foils (i.e., G, GLC24, GHC24, and GHC60) and dried at 60°C for 24 h in an oven. The prepared electrodes were labeled NMOG-G, NMOG-GLC24, NMOG-GHC24, and NMOG-GHC60, respectively. The active material loading on the electrodes is 2 mg cm^-2^.

### Materials characterization

Structural characterization was performed using X-ray diffraction (XRD; Bruker, Billerica, MA, USA) with Cu-Kα radiation (λ = 0.1542 nm) in the range of 2θ = 5–80° with a step width of 0.016°. Fourier transform infrared spectrometry (FTIR, ABB Bomem System-KBr) technique was also performed in frequency range of 400 to 4000 cm^−1^ to identify the molecular bonding. The microstructure and morphology of the as-prepared electrode materials and graphite foil substrate were examined using a scanning electron microscope (SEM; model MAIA3, TESCAN, Brno, Czechia).

### Electrochemical investigations

The electrochemical performance of NiMoO_4_/NG nanocomposite electrodes constructed with graphite foil current collectors was investigated in a three-electrode system. The reference and counter electrodes were calomel and platinum, respectively, and the electrolyte was 1.0 M KOH. Cyclic voltammetry (CV) was carried out at voltages of 0–0.7 V at scan rates of 5–100 mVs^−1^ using a VersaSTAT 4 Potentiostat Galvanostat (Ametek, Berwyn, PA, USA). Galvanostatic charge–discharge (GCD) tests were conducted at voltages of 0–0.5 V at various current densities. Electrochemical impedance spectroscopy measurements were performed over a frequency range of 0.01 Hz to 100 KHz at a voltage amplitude of 10 mV.

## Results and discussion

### Materials characterization of electrodes

**Fig 1:**
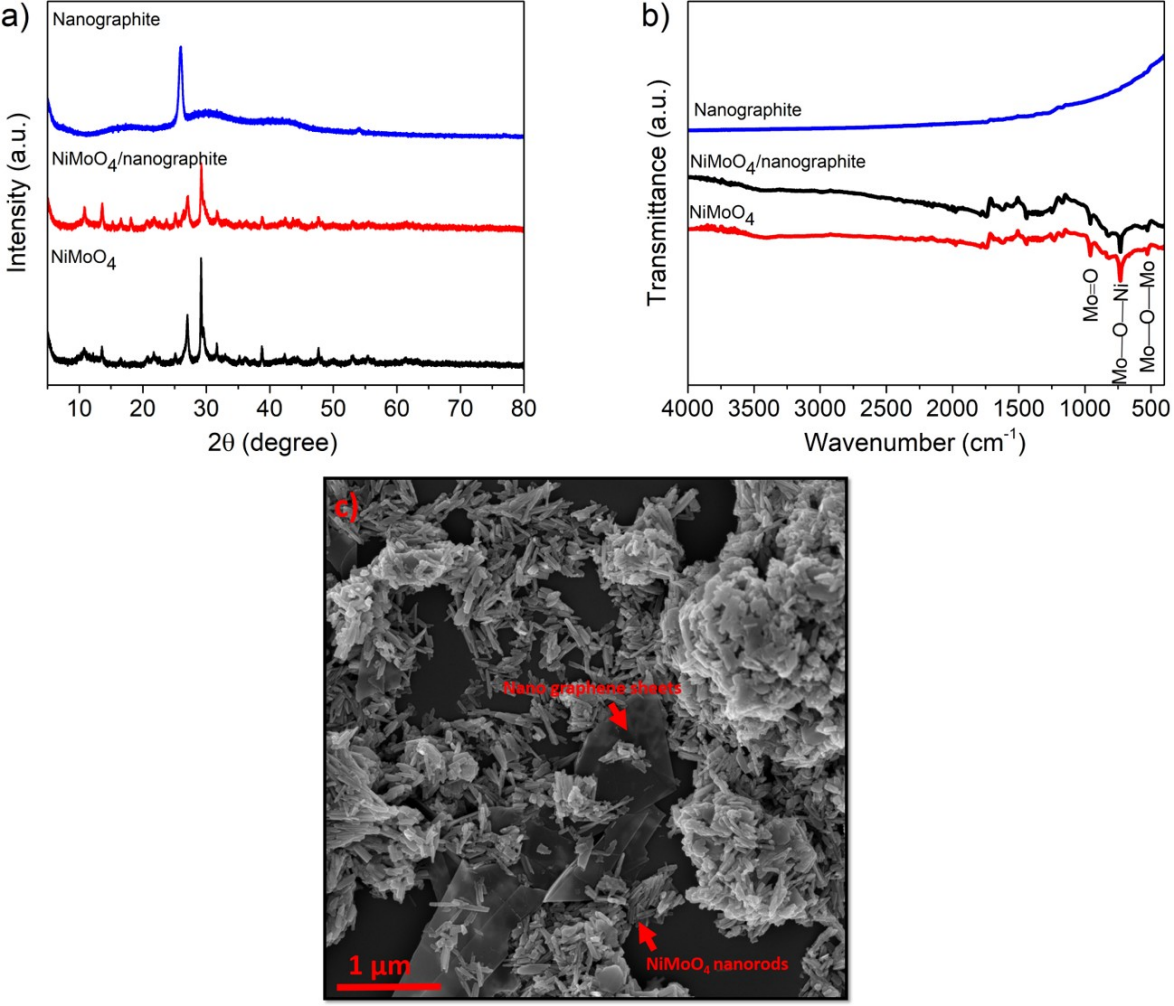
a) XRD patterns and b) FTIR spectra of the NG, NiMoO_4_ powder, and NiMoO_4_/NG nanocomposite; c) SEM images of the NiMoO_4_/NG nanocomposite.

The crystalline phase and chemical composition of the NG, NiMoO_4_, and NiMoO_4_/NG nanocomposite were characterized using powder XRD and their patterns are depicted in Fig 1A. As can be seen from the XRD patterns, the NiMoO_4_/NG sample had diffraction peaks at 2θ of 11.3°, 14.2°, 26.8°, 29.9°, 39.1° and 48.2°, corresponding to those of NiMoO_4_ [22]. In addition, the peak at 2θ of 26.8° for the NiMoO_4_/NG sample had broadened, which was related to nanographene. Moreover, the sharp, high-intensity peaks show that the NiMoO_4_/NG nanocomposite has good crystallinity.

Fig 1B displays the FT-IR spectra of nanographite, NiMoO_4_ and NiMoO_4_/nanographite samples. The FTIR spectrum of nanographite have revealed no specific peak. This means that nanographite is chemically inert [23]. It can also be observed that spectra of NiMoO_4_ and NiMoO_4_/nanographite nanocomposite are similar. The bonds before 1000 cm^−1^ were corresponded to metal oxides. The characteristics bonds of NiMoO_4_ have been seen at 526 cm^−1^ and 730.9 cm^−1^, which are attributed to Mo–O–Mo and Mo–O–Ni vibrations, respectively. The bond at 958 cm^−1^ can be related to the symmetric stretching of Mo = O bond [24].

An SEM image of the microstructure of the NiMoO_4_/NG nanocomposite is depicted in Fig 1C. As can be seen, NiMoO_4_ with a nanorod morphology was attached to a nanographene sheet. The nanorods were in the size range of 40–60 nm diameter and 100–120 nm length. Furthermore, it can clearly be observed that a porous structure has formed. This nanorod morphology of NiMoO_4_ provides a larger surface area, exposing a higher number of reaction sites. Also, a high-porosity structure facilitates ion diffusion from the electrolyte to the active materials, enhancing the reaction activity and capacitance.

**Fig 2:**
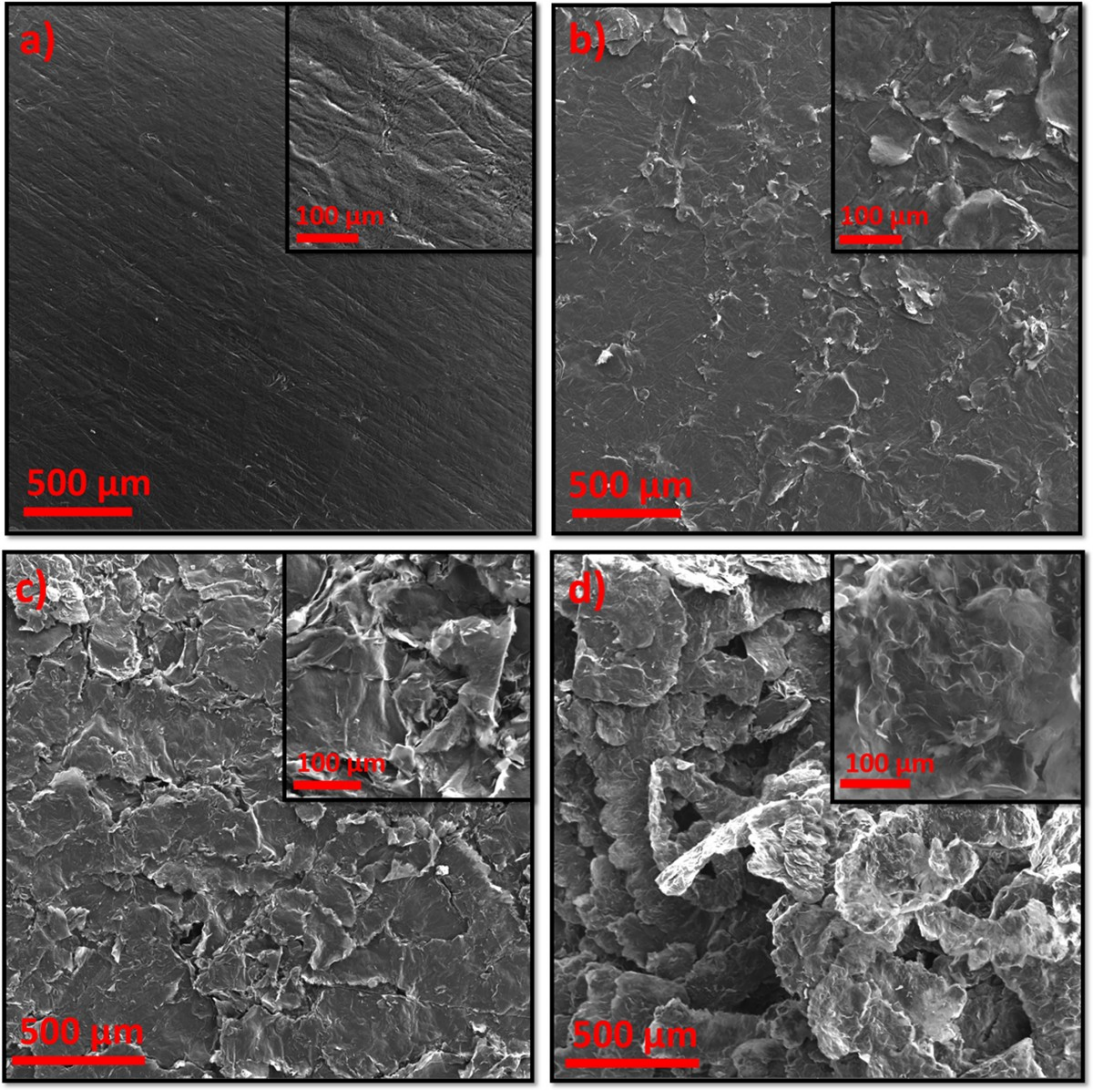
Low- and high-magnification scanning electron microscopy (SEM) images of the a) pristine G, b) GLC24, c) GHC24, and d) GHC60.

Fig 2 shows SEM images of the pristine graphite, i.e., G, and the acid-treated graphite foil substrates, i.e., GLC24, GHC24, and GHC60. Fig 2A shows that the surface of G foil is smooth, while the acid-treated foils have rough surfaces (Fig 2B–2D). The rough surfaces of the treated foils probably resulted from exfoliated graphene layers. In addition, it can be observed that the surface areas of the graphite foil substrates treated with acid are much larger than that of G. In the high-resolution inset image in Fig 2A, grooves can be seen on the smooth surface of G. In Fig 2B showing GLC24 foil, treated with low-concentration acid, the small grooves in G appear to have become cracks, and some thick exfoliated layers of graphite have appeared. Also, as seen in Fig 2C showing GHC24 foil, a rough surface with exfoliated layers was produced during acid washing. These exfoliated layers provide suitable sites in which to embed NiMoO_4_ particles. Furthermore, an increase in the extent of edges and surface defects is also observed. It is believed that the rough surface of GHC24 reduces the problem of material sloughing off from the surface. As seen in Fig 2D showing GHC60, the exfoliation of the graphite surface is obvious and a large number of graphite flakes have formed on the surface of graphite foil substrate. The graphite flakes are 50–490 μm wide and appear thin with semi-transparent layers. This indicates that the interlayer spacing has increased and that the graphite flakes consist of single or a few layers of graphene. The penetration of the acid into the defective sites has therefore led to local delamination [25]. It can be concluded that, depending on the acid concentration and temperature of the treatment, the amount of exfoliation on the surface of the graphite foil substrate will vary. Treatment with higher-concentration acid and at a higher temperature results in more exfoliation. In fact, more exfoliation and a rougher surface of the graphite foil substrate, which are obtained in treatment with higher concentration acid (Fig 2C and 2D) couldprevent the material from disintegrating easily. As a consequenceit would improve the cycling performance in supercapacitors in which the foil is used.

### Electrochemical investigation of the electrodes

**Fig 3:**
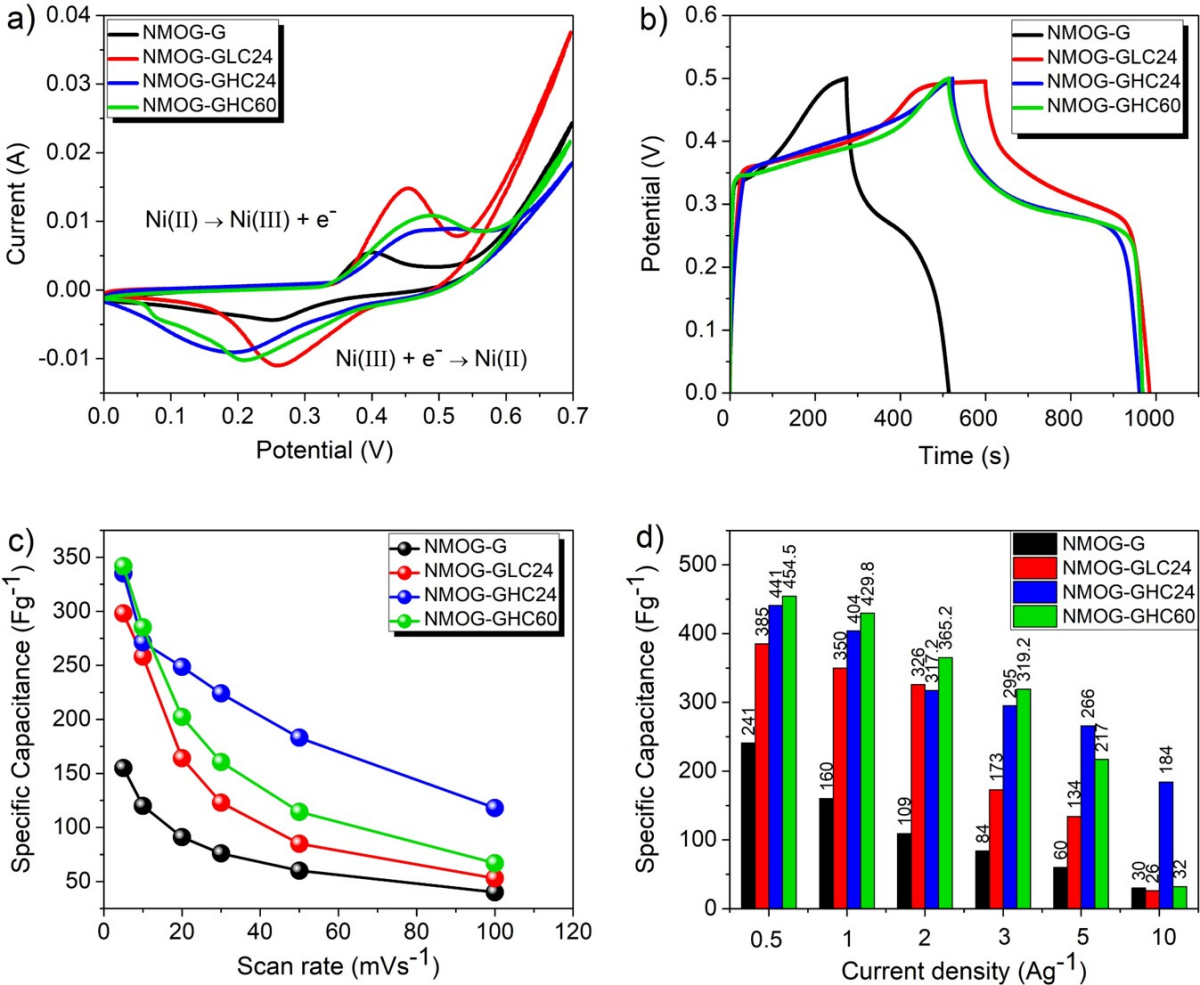
a) Cyclic voltammetry (CV) curves at a scan rate of 5 mVs^−1^; b) galvanostatic charge–discharge (GCD) curves at a current density of 0.5 Ag^–1^; c) specific capacitance versus scan rate; and d) specific capacitance versus current density for NMOG-G, NMOG-GLC24, NMOG-GHC24, and NMOG-GHC60 electrodes.

Fig 3A shows the cyclic voltammetry (CV) curves of NMOG-G, NMOG-GLC24, NMOG-GHC24, and NMOG-GHC60 electrodes at a scan rate of 5 mV s^−1^ and over a voltage range of 0–0.7 V. All CV curves display two redox peaks, corresponding to the reversible redox reactions of Ni^2+^↔ Ni^3+^+e^−^ [16, 26]. This confirms the Faradaic behavior of NiMoO_4_ nanorods. According to the literature [4, 27, 28], molybdenum is not involved in the redox reactions, but rather enhances the electrical conductivity of nickel molybdate. Moreover, the CV curves of the electrodes with pre-treated graphite foil substrate indicate a larger enclosed area than that of the NMOG-G electrode with pristine G substrate. This indicates that electrodes with treated graphite foil substrate have a higher charge storage capacity, implying that acid treatment improved their electrochemical performance. This might be attributed to the larger surface area obtained in GLC24, GHC24, and GHC60 substrates during acid treatment, and to the better adhesion of active material to the pretreated current collector.

Fig 3B shows the galvanostatic charge–discharge (GCD) profiles of the NMOG-G, NMOG-GLC24, NMOG-GHC24, and NMOG-GHC60 electrodes at a current density of 0.5 Ag^–1^ and over a voltage range of 0–0.5 V. As seen in Fig 3B, the GCD profiles of all samples are nonlinear with a pair of voltage plateaus; this indicates the Faradaic battery-type characteristics of Ni^2+^/Ni^3+^ in these electrodes [28]. The GCD curves also indicate that electrodes with pre-treated graphite foil substrate have a higher specific capacitance than does the NMOG-G electrode with its pristine G substrate.

The specific capacitance as a function of the scan rate is plotted in Fig 3C. Fig 3C shows that NMOG-GLC24, NMOG-GHC24, and NMOG-GHC60 electrodes have specific capacitances of 298, 335, and 341.85 Fg^−1^, respectively, at a scan rate of 5 mVs^−1^, capacitances significantly higher than that of the NMOG-G electrode with a specific capacitance of 161 Fg^−1^ at a similar scan rate. It can be concluded that the acid-treated graphite foil substrate allows better adhesion of active material to the current collector, resulting in higher capacitance. In addition, it should be noted that the specific capacitance decreases when increasing the scan rate from 5 to 100 mVs^−1^ (see Fig 3C). At a higher scan rate, a faster redox reaction happens, which reduces the electrolyte ion diffusion into the active material and consequently lowers the capacitance [26, 29].

The specific capacitance of the electrodes as a function of current density is plotted in Fig 3D, which shows that the specific capacitance decreases in all electrodes with increasing current density. Actually, at higher current densities, intercalation of ions into the inner active sites becomes more difficult, reducing the specific capacitance. Moreover, the mechanical expansion of NiMoO_4_ during the ion intercalation/deintercalation process can cause some NiMoO_4_ to slough off, lowering the capacitance [26, 27, 30]. At a current density of 0.5 Ag^−1^, the NMOG-GHC60 and NMOG-GHC24 electrodes display specific capacitances of 454.5 and 441 Fg^−1^, respectively, higher than those of the NMOG-GLC24 and NMOG-G electrodes, with specific capacitances of 385 and 241 Fg^−1^, respectively. Moreover, observe in Fig 3D that, compared with the other electrodes, the NMOG-GHC24 electrode experiences the smallest drop in capacitance between the current densities of 0.5 and 10 Ag^−1^, from 441 Fg^−1^ to 184 Fg^−1^, respectively. Note that the specific capacitance of the NMOG-GHC24 and NMOG-GHC60 electrodes versus current density displays a similar trend, except at the highest current density (i.e., 10 Ag^−1^). The specific capacitance of the NMOG-GHC60 electrode drops significantly at the highest current density of 10 Ag^−1^, while the specific capacitance of the NMOG-GHC24 electrode at a current density of 0.5 Ag^−1^ is about 42% of the value at 10 Ag^−1^. This indicates that the NMOG-GHC24 electrode has a good rate capability, possibly because NMOG-GHC24 experiences only moderate exfoliation of the graphite foil surface (Fig 2C), while NMOG-GHC60 experiences strong exfoliation of graphite, which might become loose (Fig 2D). Therefore, for the NMOG-GHC60 electrode at a high current density of 10 Ag^−1^, active material can easily become detached from the graphite foil current collector, while for the NMOG-GHC24 electrode, active material is probably retained on the substrate. That is why a reasonable rate capability is observed for the NMOG-GHC24 electrode, whereas a significant reduction in specific capacitance occurred at 10 Ag^−1^ for the NMOG-GHC60 electrode due to material sloughing off.

**Fig 4:**
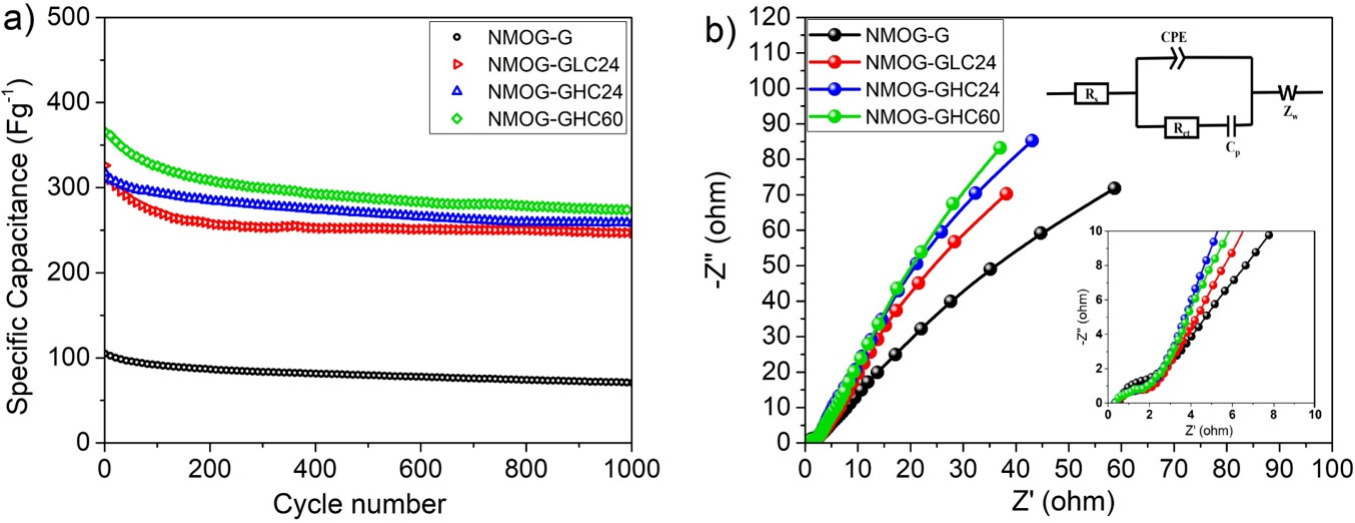
a) Cyclic stability at 2 Ag^–1^ and b) Nyquist plots of the NMOG-G, NMOG-GLC24, NMOG-GHC24, and NMOG-GHC60 electrodes.

Fig 4A shows the cyclic stability curves of the NMOG-G, NMOG-GLC24, NMOG-GHC24, and NMOG-GHC60 electrodes under continuous charge and discharge at a current density of 2 Ag^–1^ for 1000 cycles. Fig 4A shows that in the initial charge–discharge cycles, the specific capacities gradually decrease, after which they stabilize at a certain value. Finally, after 1000 cycles, the graphs indicate retention levels of 67.2%, 76.7%, 82.7%, and 75% for the NMOG-G, NMOG-GLC24, NMOG-GHC24, and NMOG-GHC60 electrodes, respectively. This means that the NMOG-GLC24, NMOG-GHC24, and NMOG-GHC60 electrodes are more stable than the NMOG-G electrode. It is also clear that a certain amount of NMOG material has become detached from the untreated graphite foil substrate when immersed in KOH (i.e., cycling), while detachment is not seen in electrodes with treated graphite NMOG material.

In general, this implies that the porous structure and rough surface produced in the acid-treated graphite foil substrate favored the stability of cycling performance. In fact, in acid-treated graphite foil substrate, NiMoO_4_ nanoparticles are well embedded in the graphite matrix and between the graphite flake layers, which enhances the adhesion between NiMoO_4_ and the graphite foil substrate. This good adhesion between the current collector and active material gives structural stability during volume expansion, which subsequently prevents the sloughing off of the active material during cycling [27]. Moreover, the NMOG-GHC24 electrode has the highest stability over 1000 cycles of all the electrodes examined here. This supports the discussion in section 3.2 that the most appropriate treatment is that of the NMOG-GHC24 electrode, in which the adhesion between the graphite substrate and active material is sufficient.

Fig 4B shows the Nyquist plots of the NMOG-G, NMOG-GLC24, NMOG-GHC24, and NMOG-GHC60 electrodes over the range from 0.01 Hz to 100 KHz and at a potential of 10 mV. It can be seen that the intercepts of Nyquist plots on the x-axis are 0.56, 0.42, 0.33, and 0.36 Ω, respectively, for the NMOG-G, NMOG-GLC24, NMOG-GHC24, and NMOG-GHC60 electrodes, which correspond to the internal resistance at high frequency (see the inset in Fig 4B) [31]. The NMOG-GHC24 and NMOG-GHC60 electrodes exhibit lower internal resistances than do the NMOG-GLC24 and NMOG-G electrodes. The low internal resistances of the NMOG-GHC24 and NMOG-GHC60 electrodes are probably attributable to more efficient interfacial contact between the NMOG nanoparticles and the porous graphite foil substrates [26, 27]. This means that the GHC24 and GHC60 foils provide better adhesion for the active material to the substrate. In the high-frequency region, the Nyquist plots of all electrodes exhibit negligible semicircles, which correspond to a low charge transfer resistance (R_ct_) [26]. The low R_ct_ is related to the fast Faradaic reactions of the NiMoO_4_ nanorods together with the high electrical conductivity of the NG sheets [32, 33]. In the low-frequency region, both the NMOG-GHC24 and NMOG-GHC60 electrodes also display the highest straight line slopes, compared with those of the other investigated electrodes. This is attributable to a low ion diffusion resistance, which results in a low Warburg resistance (Z_W_) [34, 35]. Thus, the Warburg impedance (Z_W_) and the equivalent series resistance (R_s_) are in series with CPE and C_p_. Since the redox electrochemical reaction is controlled by the kinetics of charge transfer at the electrode–electrolyte interface, C_p_ is connected in series with the charge transfer resistor (R_ct_) [36]. The equivalent circuit is illustreated in Fig 4B.

It can be stated that the GHC24 and GHC60 graphite foil substrates with their porous structure allow the electrolyte ions to be transferred into the electrode more readily. This suggests that graphite foil substrates treated with high-concentration acid contain enough porosity, which favors electrolyte diffusion into the inner regions [32, 37]. Thus, during repeated charge–discharge cycles, access of electrolyte ions to the interior layers of the graphite flakes is facilitated, so more surfaces become involved in electrochemical reactions, improving the capacitance. It can be concluded that GHC24 and GHC60 current collectors provide a low diffusion resistance that could significantly improve the electronic/ionic conductivities [18].

## Conclusion

This work investigated graphite foil substrate as a current collector for NiMoO_4_/NG nanocomposite in supercapacitors. NiMoO_4_/NG was successfully synthesized by means of precipitation on graphite foil substrate. Various treatments were performed on graphite foil substrate before it was coated with NiMoO_4_/NG. The electrode treated with high-concentration acid at 24°C (i.e., NMOG-GHC24) displayed significantly better electrochemical performance than did the untreated electrode. This treated electrode displayed a substantial increase in specific capacitance as well as a small drop in specific capacitance with increasing current density. The NMOG-GHC24 electrode had specific capacitances of 441 and 184 Fg^−1^ at current densities of 10 and 0.5 Ag^−1^, respectively. Moreover, the NMOG-GHC24 electrode had a stability retention of 82.7% over 1000 cycles at a current density of 2 Ag^–1^. Surface characterization of graphite foil substrate showed that treatment with high-concentration acid at 24°C produced suitable porosity and roughness on the surface of the graphite foil substrate. This increased the adhesion of NiMoO_4_/NG to the current collector, and thus reducing the sloughing off the electrode material during frequent charge–discharge cycles, consequently enhancing the electrochemical performance.
